# Seeing us Beyond the Cuts: Young Women’s Perspectives on the Meaning of their Non-Suicidal Self-Harm

**DOI:** 10.1007/s40653-025-00786-y

**Published:** 2025-10-27

**Authors:** Agat Sold, Tehila Refaeli

**Affiliations:** 1 The Charlotte Jack Spitzer Department of Social Work, Ben-Gurion University of the Negev, 653, Beer-Sheva, 8410501 Israel; 2 Department of public health, Ben-Gurion University of the Negev, 653, Beer-Sheva, 8410501 Israel

**Keywords:** Non-suicidal self-harm, Agency, Qualitative research, Coping strategy, Sense of control, Compulsive urge

## Abstract

The recently increasing discourse on agency among young women refers to their ability to feel in control of their bodies and their environments. However, qualitative exploration of agency among young women engaging in non-suicidal self-harm (NSSI) remains scarce. This study employed a qualitative phenomenological approach with this population, utilizing semi-structured interviews with sixteen young women aged 18–26 who had previously engaged in NSSI. The interviews were analyzed using thematic analysis. The analysis yielded two overarching themes: (1) Reasons for NSSI and (2) The Dynamics of NSSI. The first theme, Reasons for NSSI, includes six sub-themes: Using NSSI as a tool for coping, the need for control, self-punishment, the need to feel, a sense of helplessness, and using NSSI as a form of communication. The second theme, The Dynamics of NSSI, comprises three sub-themes: Efforts to hide NSSI, the role of social media in learning and connecting around NSSI, and the development of a compulsive urge to NSSI. Together, these themes illuminate both the meaning participants attributed to NSSI and the ways in which the behavior developed and was sustained. In contrast to previous research that emphasizes the negative aspects of NSSI, the current findings reveal multifaceted perspectives on this behavior. Specifically, they underscore the importance of recognizing the agency these young women demonstrate while simultaneously acknowledging NSSI’s potentially harmful aspects. Therefore, addressing NSSI requires recognizing its nuances and supporting young women who experience it. Further implications are discussed below.

## Introduction

According to the American Psychiatric Association (DSM-5; Diagnostic Manual of Mental Disorders, 2022), non-suicidal self-harm (NSSI; also known as self-injury or self-mutilation) is a behavior in which a person intentionally harms their body by cutting the skin, opening healing wounds, inserting objects under the skin, causing skin burns, self-biting, hair pulling, self-burning (e.g., cigarettes on the body) and/or banging the head and self-scratching, all without suicidal intent.

Despite extensive epidemiological and quantitative research on NSSI, growing scholarly attention has increasingly focused on understanding this behavior through the lived experiences of those who engage in it. Given that NSSI prevalence is particularly elevated among young women (Beckman et al., 2019; Bresin & Schoenleber, 2015; ; Stänicke et al., 2018), researchers have increasingly called for centering the voices of young women who engage in self-harm (Curtis, 2018; Long, 2018; Stänicke et al., 2018). The present study responds to this need by adopting a phenomenological approach to examine how young women retrospectively understand the reasons behind their NSSI use and the dynamics associated with this behavior.

### Epidemiology and Risk Factors of NSSI

NSSI has increased globally in recent years, with some researchers characterizing this phenomenon as epidemic (Fox et al., 2018; Laghi et al., 2016; Sornberger et al., 2012). In the United States, the lifetime prevalence of NSSI among adults is estimated at 5.9%, with a 12-month prevalence of 0.9%, based on a random-digit dialing survey of the general population, which, while designed to reflect the population, is not fully representative (Klonsky , 2011).

NSSI typically begins in early adolescence (Muehlenkamp et al., 2019; Jacobson et al., 2008; Nearchou, 2022), with prevalence rates of 17.2% among adolescents, declining to 13.4% among young adults (Swannell et al., 2014). Notably, young women demonstrate higher NSSI rates than young men, with some studies reporting up to 1.5 times greater prevalence (Beckman et al., 2019; Bresin & Schoenleber, 2015; Stänicke et al., 2018). Studies also suggest women typically injure themselves by cutting with razors, knives, or fingernails (Møhl et al., 2014; Sornberger et al., 2012).

Beyond demographic patterns, NSSI occurs frequently among individuals who experienced childhood trauma (McMahon et al., 2018). Indeed, young women (aged 16–33) who engage in self-harm commonly report early life trauma (Curtis, 2018; Gur, 2018; Stänicke, 2021), with sexual assault being particularly prominent (Curtis, 2018; Herman, 2015; Gur, 2018).

Although absence of suicidal intent constitutes a diagnostic criterion for NSSI, research demonstrates a significant correlation between NSSI and subsequent suicide attempts (Beckman et al., 2019; Glenn & Klonsky, 2013; Muehlenkamp et al., 2019), with NSSI serving as a substantial risk factor for suicidal ideation and behaviors (Gómez et al., 2015; Gulbas et al., 2015). Nock et al. (2006) proposed that individuals who engage in self-harm develop increased pain tolerance and reduced fear of injury, thereby lowering psychological barriers to suicidal behavior and potentially facilitating the transition from self-harm to suicide attempts. Additionally, repeated NSSI may cause more severe physical damage than intended, potentially resulting in infections, necessitating medical intervention, and sometimes leading to permanent scarring (Himelein-Wachowiak et al., 2022).

### Reasons for NSSI and the Accompanying Dynamics

Researchers have offered diverse explanations for NSSI occurrence over the years (Emerson, 1913; Nock, 2010; Suyemoto, 1998). Initially, NSSI was viewed as an unacceptable method for managing psychological distress and even as pathological deviance (Cameron, 2019; Gunnarsson, 2021). Early conceptualizations suggested that young women engaging in NSSI lacked adequate social and interpersonal skills for regulating emotional pain in socially acceptable ways (Cameron, 2019). Consequently, self-harm was understood as non-verbal expression of psychological distress (Adshead, 2010; Nock, 2010), with the physical injury serving to regulate emotions and “communicate” them to others (Adshead, 2010; Stänicke et al., 2018).

However, more recent qualitative studies, based on the voices of young women themselves, offer additional and even contradicting explanations (Curtis, 2018; Gulbas et al., 2015; Stänicke, 2021). These findings indicate that self-harm enables young women to experience sensation amid emotional numbness, provides a sense of control, offers relief from emotional pain, and serves as self-punishment (Curtis, 2018; Gulbas et al., 2015; Stänicke, 2021). These studies highlight that quantitative methods often fail to capture the depth and complexity of the lived experience of self-harm.

Beyond exploring motivations for self-harm, research examines various dynamics accompanying this behavior. In practice, young women who self-harm typically conceal their behavior due to guilt, shame, and social unacceptability (Davis & Lewis, 2019; Gunnarsson, 2021), often struggling alone without seeking help. Nevertheless, some young women (aged 16–33) report receiving messages from clinicians that their self-harm represents manipulative attention-seeking behavior (Curtis, 2016, 2018). Similarly, while some studies characterize NSSI as attention-seeking (Nock, 2010; Whitlock, 2009), others identify concealment as the primary dynamic, driven by guilt and shame (Davis & Lewis, 2019; Gunnarsson, 2021; Stänicke, 2021).

Another critical dynamic is that repeated NSSI may lead to uncontrollable, obsessive urges to self-harm over time (Curtis, 2018; Himelein-Wachowiak et al., 2022; Miller et al., 2021), with individuals attempting cessation reporting persistent urges despite their desire to stop (Stänicke et al., 2018). The literature remains divided regarding whether NSSI exhibits addictive characteristics, though various studies note similarities to addiction symptoms, including uncontrollable urges and cessation difficulties (Himelein-Wachowiak et al., 2022).

Previous qualitative studies involving women who self-harm often included participants who also engaged in suicidal behavior (Curtis, 2018) or examined mixed-gender samples (Davis & Lewis, 2019; Stänicke et al., 2018). Understanding young women’s motivations for NSSI, particularly among those without suicidal intent, remains important (Curtis, 2018). Given that NSSI can be easily triggered, we interviewed young women who had ceased self-harming, as retrospective reflection may provide deeper insight into the behavior’s meaning and dynamics.

#### *Theoretical Perspective*

This study was grounded in the theoretical perspective of agency to explore NSSI behaviors among young women. The term agency regards girls and young women as those who understand and are aware of the social constructions in which they live. Unlike previous studies that looked at them as victims, discourses based on agency tend to see girls as active, powerful, influential, and willing to succeed in the face of challenges (Krumer-Nevo et al., 2015; Mitchell & Reid-Walsh, 2009). The term agency also emphasizes the connection to the physical body, as through it girls can create a sense of control over their lives and environments (Colle et al., 2020).

Despite the relevance of agency theory to NSSI, only one previous study has examined this connection. This quantitative investigation involved twenty adults with borderline personality disorder, aged 19 to 49, nine of whom had engaged in self-harm. Findings revealed that self-harm actions (such as cutting) constituted a strategy that enabled participants to feel empowered and in control, thereby expressing their agency (Colle et al., 2020). However, to the best of our knowledge, the current study is the second to focus on the examination of agency in relation to self-harm, and the first to focus exclusively on young women.

While focusing on agency, it is essential to consider how gender norms and socialization shape young women’s experiences and expressions of agency. Many women develop within contexts that constrain emotional expression and personal autonomy (Ikävalko & Kantola, 2017), factors that may influence how they relate to their bodies and manage distress. Given that adolescent girls and young women demonstrate the highest NSSI rates among demographic groups (McMahon et al., 2018; Curtis, 2018), examining this population enables deeper exploration of how NSSI may function as a gendered form of coping, resistance, or self-regulation. This perspective complements the agency framework and underscores the importance of understanding NSSI within broader social structures that shape young women’s lived experiences.

#### *Study Goals*

This study adopted the perspective of agency to explore the reasons for and dynamic of NSSI among young women, as perceived by young women who had self-harmed in the past without suicidal intentions. The study aimed to enrich the existing literature by asking: (1) What function did NSSI have for the young women? (2) What aspects characterized the period of self-harm?

## Method

The present study employed a qualitative phenomenological research design, aimed at understanding the lived experiences and the personal meanings that young women attribute to their past engagement in NSSI. This approach seeks to uncover how individuals make sense of a specific phenomenon from their own perspective, allowing for a rich, in-depth exploration of emotional, relational, and social dynamics (Giorgi, 2009). A phenomenological lens was chosen to access participants’ subjective narratives and to better understand the underlying meanings and motivations behind NSSI as described in their own words.

### Participants and Process

The sample was a criterion purposeful sample (Patton, 2002). That is, participants were not randomly chosen but an active search was made for young women who followed the inclusion criteria: prior NSSI experience, current cessation of self-harm behaviors, and ages ranging from 18 to 30 years. A recruitment notice was circulated via various social networks. In addition, we reached out to professionals working in not-for-profit organizations providing social services to young women, asking if they knew women who had dealt with NSSI in the past and were interested in sharing their story. In both cases, prospective participants provided their contact information.

A screening interview was first conducted by phone to describe the study aims and an effort was made to prevent harm to potential participants by ensuring they were no longer self-harming before setting the final interview. That is, each young woman confirmed that she was not currently harming herself with NSSI nor with other behaviors (e.g., using addictive substances or eating disorders). The screening interview also confirmed that the participants met all the inclusion criteria (were over the age of 18; were no longer harming themselves; and that harming themselves had not been with suicidal intent).

After the screening interview, one of the researchers met with each participant either face-to-face at a location convenient for the participant or via Zoom for the full interview. Seven of the sixteen interviews were conducted via Zoom, while the remaining nine were conducted in person. The online interviews were comparable in both duration and depth to the face-to-face interview.

Semi-structured in-depth interviews were conducted, in which the young women were asked to describe themselves freely, the reasons for their self-harm, the meaning they ascribe to NSSI and the dynamic that was related to the carrying out of NSSI. Interviews ranged from 60 to 90 min in duration. Data collection continued until theoretical saturation was reached, that is, when core categories had been thoroughly developed and subsequent interviews yielded no novel insights or emergent themes (Nascimento et al., 2018). This iterative approach guided our sample size determination, adhering to established qualitative research methodological standards (Guba & Lincoln, 1994; Morse, 2011).

The sample included 16 young Israeli women, aged 18–26 years, who had carried out NSSI in their past as adolescents and young but had stopped (here from eight months to six years ago). More than a third (37.5%) completed 12 years of schooling without high school diploma, and a quarter were undergraduate students. Most were single (68.75%) and secular (87.5%). Nearly half (43.75%) had experienced sexual assault, with the perpetrator not clearly identified in most cases. The remaining participants described other potentially traumatic experiences, including prolonged social exclusion and ostracism, Experiencing verbal and physical abuse from parents, exposed to interparental violence, and loss of close family members. Notably, several participants reported experiencing multiple traumatic events. Descriptive characteristics of the participants are presented in Table 1.

**Table 1:** Descriptive characteristics of study participants*

Pseudonym	Age	Family Status	Religion Status	Education	Current Occupation	Experienced Sexual Assault
Avigail	18	Single	Secular	12 years, without HS** diploma	Unemployment	No
Sarah	21	In Relationship	Secular	12 years and partial HS diploma	Military service (volunteer track)	Yes
Rebecca	26	Single	Secular	Undergraduate student	Undergraduate student	No
Rachel	25	Single	Secular	12 years of school, without HS diploma	Studying for a diploma	Yes
Judith	19	Relationship	Secular	12 years and partial HS diploma	National Service	No
Dvora	22	Single	Secular	12 years of school without HS diploma	Working	No
Hagar	21	Single	Secular	12 years of school, without HS diploma	Unemployed	Yes
Eve	25	Single	Traditional	Undergraduate student	Undergraduate student	No
Zehava	24	Relationship	Secular	12 years of school, without HS diploma	Undergraduate student	No
Hava	22	Relationship	Secular	Undergraduate student	Undergraduate student	Yes
Lea	20	Single	Secular	12 years of study and HS diploma	Military service	Yes
Maya	23	Single	Secular	12 years of school, without HS diploma	Working	Yes
Batya	24	Single	Religious	12 years of school, without HS diploma	After National Service	No
Efrat	25	Relationship	Secular	Undergraduate student	Undergraduate student	No
Ester	20	Single	Secular	12 yearsof school and partial HS diploma	National Service	Yes
Shlomit	26	Single	Secular	Bachelor’s Degree	Working	No

* All the names are pseudonyms

** HS = High School

### Data Analysis

Interviews were fully transcribed and analyzed using MAXQDA software. Thematic analyses was used assuming that recurring themes would emerge from participants’ narratives (Braun & Clarke, 2006). The analysis was performed in several stages. The first entailed a holistic reading of all interviews to gain awareness of each story and the range of experiences of the participants. Then, the main themes of each interview were individually coded and collected. Lastly, all recurring themes were grouped into meaning units (Clarke & Braun, 2017). The analysis was initially conducted by the first author, who independently coded all interview transcripts and identified preliminary themes and subthemes. The second author then reviewed all coded data, themes, and supporting evidence, providing critical feedback and alternative interpretations where appropriate. Discrepancies in coding and theme development were resolved through collaborative discussion between both authors, with particular attention to ensuring that themes accurately reflected participants’ voices and experiences.

To promote trustworthiness and rigor in the analytic process, several strategies were employed (Lincoln & Guba, 1985; Morrow, 2005). Following each interview, the first author wrote reflective summaries capturing impressions, emotional reactions, and contextual elements that could not be transcribed (e.g., body language, pauses, tone). This supplementary data informed the analysis process to ensure interpretations remained consistent with participants’ intended meanings. Additionally, data collection continued until theoretical saturation was achieved (Nascimento et al., 2018). Finally, an iterative process of independent analysis followed by collaborative review enhanced credibility and trustworthiness. Discrepancies in coding and theme development were resolved through collaborative discussion, with final themes established through consensus. Both authors confirmed that the thematic structure adequately captured the depth and complexity of participants’ narratives.

### Ethical Considerations

This study was approved by the Research Ethics Committee at the researchers’ university. All participants had been inactive in NSSI for at least six months to reduce the likelihood that the interview would trigger self-harm. Participants provided their written consent to participate and were informed that they could stop the interview or skip questions as they wished. At the end of the interviews, participants were invited to contact the researcher should any emotional distress arise afterward. As shame and self-blame are common among those who conduct self-harm (Cameron, 2019; Curtis, 2018), added care was taken to conceal participants’ details.

## Results

The analysis yielded two overarching themes: (1) Reasons for NSSI and (2) The Dynamics of NSSI. Each theme encompasses several sub-themes that reflect participants’ lived experiences. The first theme, Reasons for NSSI, includes six sub-themes: using NSSI as a coping tool, the need for control, self-punishment, the need to feel, a sense of helplessness, and using NSSI as a form of communication. The second theme, The Dynamics of NSSI, comprises three sub-themes: efforts to hide NSSI, the role of social media in learning and connecting around NSSI, and the development of compulsive urges to self-harm. Together, these themes illuminate both the meanings participants attributed to self-injury and the ways in which the behavior developed and was sustained over time.

### Reasons for NSSI

The young women described a variety of reasons for their NSSI, allowing us to understand the role of self-harm in their lives. Each used self-harm for different reasons, some of which were intertwined. These reasons included, e.g., self-harm as a tool for coping or the need to achieve control. Below we present the women’s reasons in decreasing order of the frequency with which they were mentioned.

#### *A Tool for Coping: “This Is the Tool I Currently have”*

Most of the young women described NSSI as a means of coping. Their mental pain was often overwhelming and unbearable. Self-injury provided a distraction, diverting attention from mental to physical pain, thus offering temporary relief from an emotional storm. Most of the participants stressed that the relief was brief, only a few moments, but self-injury still gave them a chance to detach from a chaotic and unbearable inner reality. Lea said: “*It gave me some peace… like*,,* instead of thinking about everything I experienced that day […] instead of thinking about how much it hurts […] As if now my pain and my thoughts are focused on this [the NSSI]”*.

Lea’s words make it clear that self-harm served her as a coping strategy providing temporary relief from painful external and internal realities. Ester described her self-injury similarly, adding the important role of NSSI as a tool for survival:

It helps me, it helps me not to think […]it helps for exactly two minutes, but it’s something. Like this is the tool I have now, so I need to use it to survive this life.

Ester described NSSI as a coping strategy she chose at that time, with self-harm providing temporary relief from mental pain. Though causing harm in various ways, NSSI was seen as a means of survival and as their way of dealing with unbearable feelings due to past trauma of sexual assault, peer victimization, and exposure to violence and neglect in their family unit.

#### *The Need for Control: “The Knife Is Mine and the Control too”*

The young women indicated that NSSI allowed them to feel that they had control over their lives. Some described how control had been taken from them due to painful and traumatic early life experiences (e.g., sexual assault, unexpected losses such as the death of someone close), reinforcing their perceived inability to affect or change their lives. Batya described how self-injury allowed her to feel in control after losing several significant people: *“The feeling is that the knife is mine and the control too. […] I felt that I can decide; I control the location*,* number and size of the cuts”*.

Determining the location, the intensity and type of injury during self-injury allowed Batya to decide on many aspects of these injuries, giving her a sense of control. Zehava also said that self-injury gave her a sense of control when she felt threatened, particularly when she was verbally harmed by her parents:

There are times I think that […] it will provide some relief, some sense of control […] The pain that the outside world causes you is a mental pain, but the pain that you cause to yourself, you decided to cause it.

Zehava had no control over the pain inflicted by her surroundings, but NSSI allowed her to choose the physical pain she would endure. This momentarily distracted from emotional pain while restoring a sense of control over her body.

#### *Injury as self-punishment: “This Body Deserves It a Little bit”*

For some young women self-injury was a way to punish their bodies. Due to different traumas, they came to hate their bodies and wanted to harm them. Hagar described how her self-harm was primarily related to the sexual assault she had experienced:

I feel… that my body is no longer mine. Someone came and did what he wanted in this body, and now this body is not worth too much. So even if there will be signs and cuts in it…. all sorts of other things, so this body deserves it a little bit. … because eventually this body […]guilty.

Following the rape, Hagar felt worthless, guilty, and helpless. She described detaching from her body, blaming it for the assault and believing it deserved punishment.

Similar to Hagar, Eve felt guilty and unworthy in social situations. Even small arguments with friends and family caused her feelings of anger, remorse, and self-hatred. NSSI allowed her to punish herself for every incident for which she blamed herself: “*This is my way of coping… if […]… I get angry at myself; self-harm is my way of dealing with the anger*”. For Eve, NSSI was a punishment for her not fitting her expectations of herself and achieving the goals she set for herself. That is, guilt and self-criticism were the major reasons for these young women’s self-punishment. Punishing themselves with their bodies also allowed them to deal with their overwhelming emotions. For Eve, NSSI functioned as self-punishment for perceived failures to meet personal standards and goals. Guilt and self-criticism were central factors, with self-harm serving as a mechanism for managing intense emotional states.

##### Self-injury in order To Feel: “To Feel Pain Is Actually To Feel”

Several participants described emotional detachment as a trauma response to avoid overwhelming feelings. However, this protective dissociation resulted in affective numbing. NSSI enabled them to experience physical pain and, consequently, a restored ability to feel themselves as being alive.

Judith shared that she would hurt her feet so that the painful steps allowed her to feel what she couldn’t feel without them: “*I would do it with my foot […] I would say to myself*,* maybe I will do it and then every time it hurts I will feel. Every step I take I will feel*”. Presumably, after she felt disconnected and numb, this focus on the physical pain helped her feel a pain that was under her control. Similarly, Avigail said that self-injury allowed her to cope with the feeling of emptiness:

I felt something empty inside me…. so, these cuts helped me simply feel, feel something. They also help me feel warm. When the blood flowed, it was hot. And I felt like someone hugging me […] Yes, that sounds weird […] but at least I felt something.

Not only did self-injury allow Avigail to feel present, she described the warmth of blood as an embrace. With her sense of emptiness self-injury provided warmth and feeling of aliveness.

#### *Because of Helplessness: “You Feel at a dead-end”*

Although most of the participants said that they often hurt themselves out of a desire to cope, some of them sometimes also self-harmed out of a sense that they had no choice but to hurt themselves. There were times when they reflexively or compulsively harmed themselves out of deep despair without premeditation. The young women described self-harm as a response to ongoing and unbearable helplessness. Rachel, for instance, described her distress as a dead-end:

I feel helpless and don’t know what to do. This is not once in long time […] It’s actually daily distress. So part of the self-harm is the thought, the hopelessness about the future, the feeling that you no longer care about yourself[…]You feel at a dead-end with no other options than to self-harm.

Rachel described self-harm as her only way of dealing with helplessness and distress. Batya also felt helpless; it was difficult for her to grasp the contradiction that she was alive yet felt dead inside:

All the time I was sure that I wouldn’t be able to deal with the mental pain and maybe it would kill me […] I could not understand how it is possible to be hurt so much inside and you walk as usual and everything is good.

Batya felt that sometimes her inner pain was so intense that in her eyes, she and young women like her had no other option but to self-harm.

#### *Communicate my Distress: “Maybe now They Will Understand my pain”*

Contradicting the widespread assumption that self-harm is an attention-seeking behavior (Nock, 2010), this reason was mentioned rarely in this study. Only a few of the young women described self-harm as an attempt to communicate their distress to others. Note that these young women at first hid the NSSI, then later stopped hiding and tried to use the NSSI to reflect their mental state and the chaos in their lives. Avigail shared that she tried to express the extent of her distress at home: “*I started doing it for attention. I would really severely cut myself before school. … and then come to school so they [the staff at school] could see that I was in distress. It happened because my family was violent; my dad hit my mom*”. Avigail did not conceal her injuries and occasionally displayed them at school, though this was not her primary intention. Rather, self-harm served as a coping strategy for mental pain. Similarly, Rachel contended that self-harm was a cry of distress:

*It wasn’t for attention but for others to know I was in great pain*,* that I am not well. Often this occurs in front of professionals […] So*,* yes […]Self-harm can be a way to communicate this*,* maybe now they will understand my pain.*

For Rachel, NSSI was a means to get caregivers to see her distress and make them understand what she could not say. We can see that self-harm may become a means of outwardly expressing mental pain.

In summary, NSSI served multiple functions: emotional regulation, regaining perceived control, self-punishment, managing trauma-related numbness, and communicating psychological distress. Each young woman harmed herself for different reasons and sometimes for more than one. For some of them their reasons changed over time. For instance, Ester initially engaged in self-harm for emotional relief but later sought control; Rachel similarly described a change from control-seeking to responding to feelings of helplessness. These findings also underscore the dynamic and multifaceted nature of NSSI.

### The Dynamic of NSSI

The young women in this study harmed themselves repeatedly and the dynamics described below are associated with repeated self-harm.

#### *Hiding NSSI: “I Just Wanted To Hide It”*

While some young women used self-harm to communicate distress, as described above, most told in detail of the great effort needed to hide self-harm from others. Each had her specific reasons to keep this secret. For example, Efrat described how she long hid her self-injury from her parents: “*I worked very hard at hiding the self-injury from them […] It was not as if my parents were not concerned or did not notice […] I never admitted to them of harming myself”*. Efrat emphasized that concealing self-harm required effort and energy to ensure that others did not know or suspect. Rebecca shared that, although professionals and society tend to see NSSI as a means of gaining attention, she took care to hide the self-harm from everyone’s eyes and even stressed that it was her personal secret:

People think they [young women] do it so that they will get attention, that they will be taken care of, that it will be seen that it is difficult for them … “Come and see how hard it is for me, so I am cutting myself” […]. Ah, and it was not that at all.

Ester stressed that the need to conceal was not derived just from feelings of shame but also from fear that if the staff at her group-home found out they would declare her ‘mentally ill’ and she could be hospitalized against her will: “*I felt I could not tell anyone because I had been threatened with psychiatric hospitalization; I was very scared”*. The staff had found out and had threatened that if Ester self-harmed again, she would be hospitalized. Therefore, she was in constant fear that they would discover that she was continuing with NSSI.

The fear of professional misinterpretation of NSSI, such as viewing it as indicative of severe mental illness requiring psychiatric hospitalization or dismissing it as attention-seeking behavior, frequently motivated participants to conceal these behaviors. Several young women described investing considerable effort in hiding their self-harm from both healthcare providers and family members.

### Social media as a place of learning and belonging in relation to self-harm: “*The greatest place to learn and talk about it“*

The young women shared that, although they hid the self-harm from their surroundings, they felt comfortable revealing and sharing it in certain arenas of social media. Some even told that they began to harm themselves only after learning about NSSI from various media. For example, Eve told that a member of the youth movement to which she belonged revealed the issue to her, and within a few days she had already experienced it herself:

So a friend from the movement, in eighth grade, we were in a good relationship, and… I remember like I was already in the world of [the blog name], and I think I even knew her through [the blog name], and then she [the friend] told me that she was, that she was cutting, and in a day or two like… I checked it myself.

Eve’s comments underscore the fact that self-harm sometimes was learned by exposure to someone else engaged in it. This is a surprising finding given that the young women emphasized the element of hiding self-harm. This contrast can be explained by the anonymity possible in blogs and online forums.

Ester also shared that she was exposed to self-harm and learned to harm her body through an online community of friends:

They added me, like one Friday, to a Facebook group of writing and there was a lot of content of suicidality and self-harm. That’s where I was exposed to self-injury for the first time.

Ester’s words show the ease of exposure to self-harm in the network. She then explains the contagion process in the group:

Someone named (the girl’s name) wrote ‘this is the day I cut myself’, and then someone named (the girl’s name) writes to her ‘If you cut, then so will I cut’, and then it’s like a chain reaction and it’s very retarded.

It seems that exposure to others who harm themselves increases the risk that young women will try it themselves, but it also allows them to feel that they are not alone and even feel social belonging. This sense of universality allows them to feel that they are not as unusual as they usually felt in other social frameworks. Also, on social networks they are the ones who decide when and how they reveal their self-injury, another aspect that may allow them to feel more comfortable in this space.

#### *The Compulsion To self-harm: “Suddenly It Took Me over”*

While most participants initially described NSSI as a coping mechanism for overwhelming psychological distress, some reported that it subsequently developed into a compulsive behavior that became increasingly difficult to control. This compulsive quality sometimes persisted even after cessation of active self-harm, extending to the time of interview. Eve equated NSSI with addictive substances:

And there is… a sense of euphoria with the release of endorphins, like alcohol and drugs. It becomes a compulsion […]every time I do it, I feel better, and it gets easier […] I remember that there really were times when, even if I was not in some very difficult predicament, I would cut myself.

Like Eve, Avigail shared in the interview that the injury itself allowed her to disengage from everything, similar to the feeling of disconnection obtained by the use of a psychoactive substance:

It’s like the most calm drug that can be, and … it’s like bringing you such wings and you just … in your world, and you do not think about the problems.

Avigail also emphasized the pleasure and euphoria resulting from the self-harm that leads the person to experience a different reality for a few moments, similar to psychoactive substances. The NSSI allowed her to detach from the difficulties and problems and one can therefore understand why it produces an addictive effect.

Following these positive effects of NSSI described by the young women, the cessation of this behavior was hard. Batya shared what she wrote to herself while trying to stop cutting: *“This is a hard*,* painful detox. The body needs the cuts like food to eat or air to breathe. How can you hold back so much?“*. Batya suggested that the process of stopping NSSI was like withdrawal, underscoring the intense compulsion to NSSI. Her need for self-harm akin to basic physiological needs.

In summary, three key behavioral dynamics emerged regarding NSSI. First, participants frequently concealed their self-harm due to shame, guilt, and social stigma, as well as concerns about professional misinterpretation (e.g., as suicidal ideation) or social judgment (e.g., as manipulative behavior). Second, while NSSI remained hidden in their offline lives, some participants discovered online communities where they found validation and belonging. Third, although self-harm initially functioned as an emotion regulation and control strategy, the behavior often evolved into a compulsive urge that participants found increasingly difficult to stop.

## Discussion

This study examined NSSI from the retrospective vision of young women who had self-harmed in their past. The importance of this study derives from the growing demand for qualitative research on those who have carried out these behaviors (Curtis, 2016; Stänicke et al., 2018). Also, recent research has shown that this phenomenon is more common among women than men, and only very few studies have examined NSSI specifically in young women (Curtis, 2016; Bresin & Schoenleber, 2015). Here, we interviewed 16 young women, who had self-harmed in the past, to learn about their reasons for NSSI and their perception of the dynamics associated with this behavior.

Many studies have explored specific aspects of NSSI. Some indicate a relation to suicide (Gómez et al., 2015; Gulbas et al., 2015), while others see it as reducing suicide (Curtis, 2018; Stänicke et al., 2018). Some studies see NSSI as resulting from helplessness and lack of interpersonal skills (Adshead, 2010; Nock, 2010*)*, while others regard it as stemming from a desire to cope (Curtis, 2016; Long, 2018). Our findings highlight that in the experience of young women with NSSI the phenomenon consists of many non-dichotomous and complex aspects that need to be recognized.

Most of the young women in this study described NSSI as a way to cope with emotional distress, highlighting a strong internal desire to manage their experiences. This aligns with the growing discourse of agency in work with young women, which emphasizes their capacity to respond actively to adversity despite limited resources (Boomkens et al., 2021; Lynch et al., 2024; Sidle, 2019). Within this framework, Colle et al. (2020) interpreted self-harm as a purposeful act reflecting an effort to escape distress and regain control. The current study is, to our knowledge, the first to apply the lens of agency specifically to young women engaging in NSSI. Our findings support and extend Colle et al.’s (2020) interpretation, revealing several dimensions through which agency is expressed in the context of self-harm, as elaborated below.

First, NSSI was described by participants as an active strategy for coping with emotional distress. Most reported that it helped shift their focus from emotional to physical pain, offering temporary relief and enabling daily functioning. This aligns with previous studies suggesting that NSSI may reduce suicidal thoughts and behaviors (Brossard, 2018; Curtis, 2016; Miller et al., 2021). Our findings highlight the young women’s inner desire to deal with their mental pain, which they do through NSSI, an aspect that reflects their agency (Moore, 2016).

Second, we found that NSSI may also be linked to a need for control. Some participants in our study, as well as in previous research, described a lack of control as a central trigger for self-harm and some reported a history of sexual assault (Gur, 2018; Shenk et al., 2010). Others described different traumatic or negative life events. Such experiences often leave survivors feeling powerless and devalued (Herman, 2015). Through self-harm, the young women described regaining a sense of control over their bodies (see also Gomez et al., 2015; Hawton et al., 2012). The participants here referred to their ability to determine the appearance, placement, and intensity of their cuts providing a sense of the control that had been taken from them. NSSI thus became a factor that in their eyes strengthened their agency, as it helped them deal with unbearable feelings of hopelessness.

Third, some of the participants also described the need for self-punishment, in the context of sexual assault. The loss of control they experienced when sexually assaulted led some to self-blame and generated the urge to punish their body by hurting it. Self-blame is common among trauma survivors, especially survivors of sexual assault (Shir, 2018). According to Janoff-Bulman (1979), it is easier for sexually assaulted women to blame themselves and feel that they should change their behavior (e.g., clothing) rather than blame external factors beyond their control (see Shir, 2018). Self-blame can thus provide the survivors with a sense of balance and control, allowing them to adapt to life after sexual assault (Janoff-Bulman, 1992). The current findings add that NSSI may also enhance a sense of agency, as punishing their bodies allowed the young women to regain a sense of control over them.

Finally, some trauma survivors described feeling numb and that NSSI allowed them to feel, if only for just a few moments. Helplessness resulting from trauma (Herman, 2015), causes some to lose the ability to feel or makes them feel as if they are watching their life from outside (i.e., depersonalization). While this reduces the feelings of pain and anxiety accompanying trauma, staying in a state of emotional numbness impairs the quality of life and perpetuates the impact of the traumatic event (Herman, 2015). Here, the young women’s desire to break out of the sense of disengagement led to some of them using NSSI (see also Klonsky, 2007; Whitlock, 2009).

Some of the young women in our study shared that the sight of blood helped them move out of a sense of disconnection. Bar-On (2011) similarly found the sight of blood to evoke strong feelings. NSSI for this reason can be seen as another manifestation of agency. That is, young women’s experiences of strong emotional and physical sensations through NSSI enabled them to actively leave their disconnection; self-harm thus reflects their control, power and agency.

Alongside seeing NSSI as enabling a sense of coping that marks the agency of the young women, the present study’s findings also revealed the negative aspects of this behavior. Only a minority of the young women here told that their self-harm came from a position of hopelessness, out of deep despair and lack of faith in their future. This finding contrasts with studies that found hopelessness a major cause of NSSI (Gray et al., 2021; Mclaughlin et al., 1996), as well as the inability to regulate emotions (Nock, 2010; Whitlock, 2009). In these cases of NSSI as a result of hopelessness, the injury did not appear to reflect the young women’s desire to cope.

Recent research has shown that NSSI causes the release of endorphins, which foster feelings of relief and euphoria. Over time, a compulsion to regain this state may develop (Miller et al., 2021). Our findings support the possibility that NSSI can become compulsive, increasing in frequency and intensity over time in order to achieve the same effect. That is, while the needs described above may predominate in the early stages of NSSI, it may later evolve into a physical and emotional dependency similar to other forms of addiction. The inability to regulate the intensity of the injury may place young women at significant physical risk, as well as at risk of losing the sense of agency they initially experienced.

These findings highlight NSSI as a complex and dynamic behavior. In the short term, it may provide a sense of coping and control that reflects agency, but over time it may also emerge from despair and hopelessness, eventually becoming an uncontrollable urge in which agency is diminished. Thus, our findings expand on those of Colle et al. (2020), who emphasized self-harm as an expression of agency. The current study underscores the importance of recognizing young women’s agency, while also acknowledging the compulsive aspect of NSSI that can develop over time. Recognizing both elements may allow professionals and services to approach the phenomenon more holistically and, in turn, better understand the young women.

Importantly, only a few participants indicated that they used NSSI to make others aware of their mental distress. As in previous studies, most of the young women reported concealing their self-harming behavior due to feelings of shame, as this behavior is socially unacceptable (Davis & Lewis, 2019; Stänicke, 2021; Gunnarsson, 2021).

Another reason for hiding their injuries, identified in the current study, is that NSSI is often perceived by professionals as unacceptable and dangerous. Accordingly, Curtis (2018) notes that young women who self-harm often avoid seeking help due to fears of being misunderstood. However, if professionals recognize the behavior as an expression of agency while acknowledging its inherent risks, young women may feel their experience is understood more holistically. This approach may encourage disclosure and reduce shame or fear of judgment. That is, recognizing agency behind NSSI, while addressing safety concerns, may support more sensitive and effective interventions.

The Internet is known to be a prominent space where young people share their experiences of self-harm (Davis & Lewis, 2019). In the present study, participants’ use of social media revealed that they often chose how to present themselves or remained anonymous in order to feel comfortable discussing their self-harming behaviors.

There are several limitations to this study. Due to language difficulties, only young women who were fluent in Hebrew were interviewed for the study. Thus, this study does not represent the NSSI experiences of minorities or young immigrant women. In addition, while a retrospective interview with the participants may provide full and comprehensive understanding of the young women’s experiences with NSSI and be ethically safer for the participants, the participants’ memories may be mixed with their present perspective. Notably, because NSSI is a highly sensitive and stigmatized topic, participation was limited to young women who felt emotionally secure and open enough to share their experiences. The voices of those currently in distress or who did not feel safe participating may be absent from this study. Furthermore, the interviewer’s background as a social worker may have influenced interview dynamics and participants’ willingness to disclose their experiences. Finally, some of the interviews were conducted via Zoom. Although recent research suggests that online interviews can produce data of comparable quality to face-to-face interviews, slight differences in participants’ perceptions of rapport and empathy have been found (Anthony et al., 2025). It is therefore possible that the virtual format influenced certain interpersonal dynamics, even though the richness and depth of the data remained consistent.

### Conclusions and Recommendations for Practice and Research

The literature today often emphasizes the many risks involved in NSSI. Our findings emphasize that to understand in-depth the experience of those who self-harm, professionals must see the many reasons for NSSI including the young women’s desire to try to cope and regain control and agency in their lives. The issue of uncontrollable impulses that are part of the NSSI dynamic should also be addressed in interventions with young women. Many young women in the present study noted that beyond the guilt and shame that accompany this behavior, they were afraid to seek professional help for fear that professionals would interpret NSSI incorrectly and that they would be hospitalized.

The findings carry several implications for practice. Professionals should be trained to recognize the difference between NSSI and suicidal behavior, and minimize severe responses such as involuntary hospitalization to NSSI behavior. One of the populations that suffer a lot from stigmatization in mental health systems are young men and women in the emerging adulthood period, and therefore they tend to avoid services utilization (Crumb et al., 2021; Lynch et al., 2024). Thus, Acknowledging the agency of young women who engage in NSSI, even when their behavior carries risk, may encourage help-seeking, reduce fear of stigmatization, and allow for more nuanced, non-pathologizing therapeutic approaches. Creating therapeutic spaces that support open, non-judgmental dialogue may help address both the immediate distress and the longer-term needs of young women who use self-harm.

In terms of recommendations for future studies, research should be conducted collaboratively with both mental health professionals and young women experiencing NSSI to foster dialogue between these groups’ differing perceptions. Additionally, qualitative studies with diverse populations of women who have experienced self-harm (e.g., minority groups or immigrants) are needed to identify whether alternative motivations for NSSI use exist. Future studies should also examine whether young women’s perceptions of their NSSI evolve with age or compare cohorts of young women based on the temporal distance from their self-harm experiences. Another important direction for future research is to examine how agency influence the process of disengaging from NSSI, particularly given its potential to become more compulsive over time.

## Data Availability

Data available on request from the authors.
